# Efficacy of a ‘lethal house lure’ against *Culex quinquefasciatus* from Bouaké city, Côte d’Ivoire

**DOI:** 10.1186/s13071-023-05883-1

**Published:** 2023-08-28

**Authors:** Innocent Z. Tia, Antoine M. G. Barreaux, Welbeck A. Oumbouke, Alphonsine A. Koffi, Ludovic P. Ahoua Alou, Soromane Camara, Rosine Z. Wolie, Eleanore D. Sternberg, Amal Dahounto, Gregoire Y. Yapi, Matthew B. Thomas, Raphael N’Guessan

**Affiliations:** 1 Vector Control Products Evaluation Centre (VCPEC)/Institut Pierre Richet (IPR), Bouaké, Côte d’Ivoire; 2 Institut Pierre Richet (IPR)/Institut National de Santé Publique (INSP), Bouaké, Côte d’Ivoire; 3 Université Alassane Ouattara, Bouaké, Côte d’Ivoire; 4 Centre d’Entomologie Médical et Vétérinaire (CEMV), Bouaké, Côte d’Ivoire; 5 Centre de coopération internationale en recherche agronomique pour le développement (CIRAD), 34398 Montpellier, France; 6 Department of Vector Biology, Liverpool School of Tropical Medicine, Pembroke Place, Liverpool, L35 QA UK; 7 Unité de Recherche et de Pédagogie de Génétique, UFR Biosciences, Université Félix Houphouët-Boigny, Abidjan, Côte d’Ivoire; 8 Laboratoire de Bio-Mathématique et d’Estimation Forestière, Université d’Abomey Calavi, Cotonou, Bénin; 9 The University of Florida, Gainesville, USA; 10 Department of Disease Control, London School of Hygiene and Tropical Medicine, London, UK

**Keywords:** Eave tubes, *Culex quinquefasciatus*, Insecticide resistance, Bouaké

## Abstract

**Background:**

Eave tube technology is a novel method of insecticide application that uses an electrostatic coating system to boost insecticide efficacy against resistant mosquitoes. A series of previous experiments showed encouraging insecticidal effects against malaria vectors. This study was undertaken to assess the effects of the eave tube approach on other *Culicidae*, in particular *Culex quinquefasciatus*, under laboratory and semi-field conditions.

**Methods:**

Larvae of *Cx. quinquefasciatus* from Bouaké were collected and reared to adult stage, and World Health Organization (WHO) cylinder tests were performed to determine their resistance status. WHO standard 3-min cone bioassays were conducted using PermaNet 2.0 netting versus eave tube-treated inserts. To assess the transient exposure effect on *Cx. quinquefasciatus*, eave tube assay utilizing smelly socks as attractant was performed with exposure time of 30 s, 1 min, and 2 min on 10% beta-cyfluthrin-treated inserts. Residual activity of these treated inserts was then monitored over 9 months. Field tests involving release–recapture of *Cx. quinquefasciatus* within enclosures around experimental huts fitted with windows and untreated or insecticide-treated eave tubes were conducted to determine house entry preference and the impact of tubes on the survival of this species.

**Results:**

Bouaké *Cx. quinquefasciatus* displayed high resistance to three out of four classes of insecticides currently used in public health. After 3 min of exposure in cone tests, 10% beta-cyfluthrin-treated inserts induced 100% mortality in *Cx. quinquefasciatus*, whereas the long-lasting insecticidal net (LLIN) only killed 4.5%. With reduced exposure time on the eave tube insert, mortality was still 100% after 2 min, 88% after 1 min, and 44% after 30 s. Mortality following 1 h exposure on 10% beta-cyfluthrin-treated insert was > 80% continuously up to 7 months post-treatment. Data suggest that *Cx. quinquefasciatus* have a stronger preference for entering a house through the eaves than through windows. Beta-cyfluthrin-treated inserts were able to kill 51% of resistant *Cx. quinquefasciatus* released within the enclosure.

**Conclusions:**

Eave tubes are a novel method for delivery of insecticide to the house. They attract nuisance host-seeking *Cx. quinquefasciatus* mosquitoes and are as effective in controlling them as they are against pyrethroid-resistant *Anopheles gambiae*, despite the high level of resistance *Cx. quinquefasciatus* have developed.

**Graphical Abstract:**

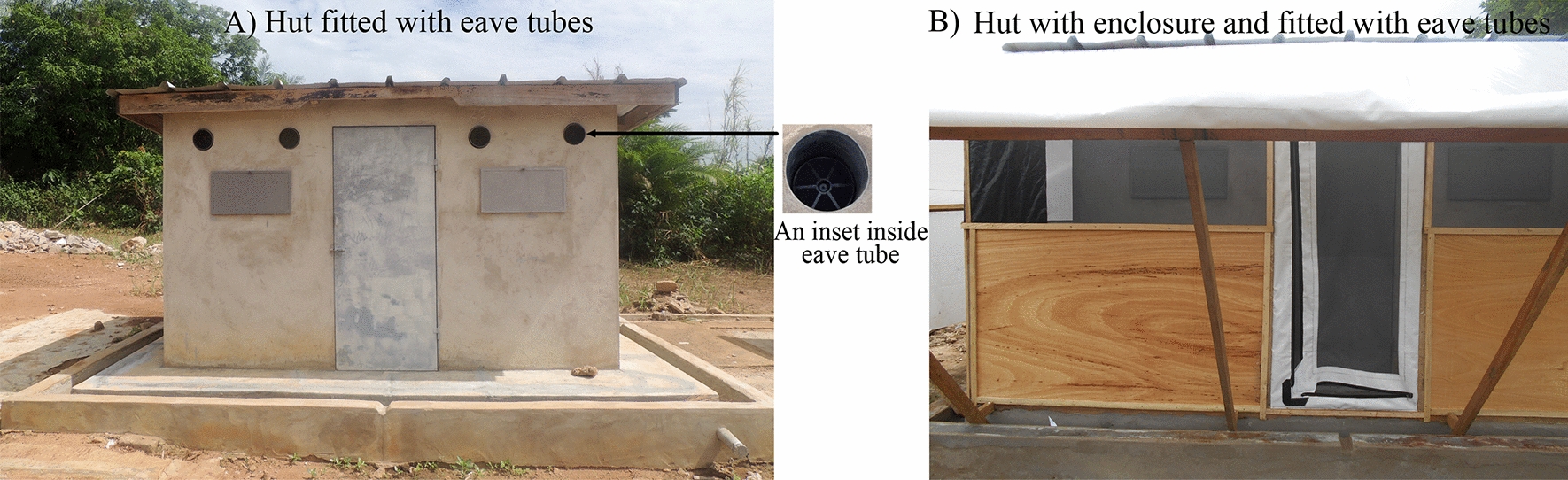

## Background

*Culex quinquefasciatus* members of the *Culex pipiens* complex are predominant in urban environments across African cities. *Culex quinquefasciatus* is a vector of lymphatic filariasis (LF) and other major arboviruses [1, 2]. LF remains a chronic disfiguring infection, with 51.4 million people infected worldwide according to the most recent report of the World Health Organization (WHO) [3]. Elimination of LF is based on mass drug administration (MDA) and vector control with public health insecticides [4]. However, the intensive use of these pesticides in urban agriculture associated with massive deployment of insecticide-treated nets has induced insecticide resistance in the three major mosquito genera [5–8]. Recently reported data have revealed high pyrethroid resistance intensity mediated by different defence mechanisms in *Cx. quinquefasciatus* populations through urban environments in major African cities [9–12]. In addition, *Culex* mosquitoes are responsible for nuisance and discomfort to the population [13]. *Culex quinquefasciatus* take blood meals from both humans and animals, and this behaviour plays an important role in the amplification and transmission of zoonotic diseases [14]. Innovative and effective vector control tools are thus needed to sustain the fight against resistant and nuisance populations of *Cx. quinquefasciatus* [15, 16].

The eave tube method has recently been used for malaria control as part of the development and testing of a house modification method that the WHO Vector Control Advisory Group (VCAG) calls a ‘lethal house lure’ [17]. The aim is to block mosquito entry while killing them as they are lured into the house and are exposed to the insecticide. This can be achieved through screening of windows and blocking eaves and adding In2Care Eave Tubes. Eave tubes are polyvinyl chloride (PVC) pipes fitted at the eave level that act like a chimney, channelling human odours and attracting mosquitoes, which enter through the tube and encounter the treated surface that kills them [18–20]. Several semi-field studies have shown that the use of these eave tubes + screening of windows decreases malaria mosquito entry and increases mosquito mortality [21, 22]. *Anopheles gambiae* mosquito entry was reduced by 60% by fitting eave tubes to West African experimental huts, with no deflection to sleepers in nearby unprotected huts, and achieving cumulative mortality of over 90% over several nights [23]. Even when considering human behaviour, the combination of screening and eave tubes has the potential to reduce mosquito entry and kill mosquitoes [24]. This was made evident in a randomized controlled trial in central Côte d'Ivoire that showed a high epidemiological impact of the technology, with a 38% decrease in the incidence of malaria [25]. The main objective of the present study was to assess the impact of this new insecticide application method on the behaviour of highly resistant *Cx. quinquefasciatus,* a nuisance mosquito often out of reach of conventional tools.

## Methods

### Mosquito collection and rearing

Mosquitoes were collected within the urban Bouaké area (Côte d’Ivoire) between January 2018 and January 2019. *Culex quinquefasciatus* larvae were sampled from polluted drains and septic tanks. Larvae were transported to the Vector Control Product Evaluation Centre (VCPEC) insectary and reared to adult stage with fish food (TetraMin™ Baby). A susceptible laboratory colony of *Cx. quinquefasciatus* (SLAB) was used as a reference.

### Insecticide susceptibility test

Susceptibility tests were performed to determine the resistance status of the wild *Cx. quinquefasciatus* using insecticide-impregnated papers. Diagnostic doses (DD) of deltamethrin (0.05%), permethrin (0.75%), cyfluthrin (0.15%), bendiocarb (0.1%), and pirimiphos-methyl (0.25%) were tested, along with a synergist (piperonyl butoxide [PBO]), and the intensity of resistance (5 × and 10 × DD) was determined. A total of 100 ± 10 non-blood-fed females (3–5 days old) per concentration were tested. Mosquitoes were exposed for 1 h and the mortality rate assessed 24 h post-exposure.

### The eave tube bioassay

**Fig. 1:**
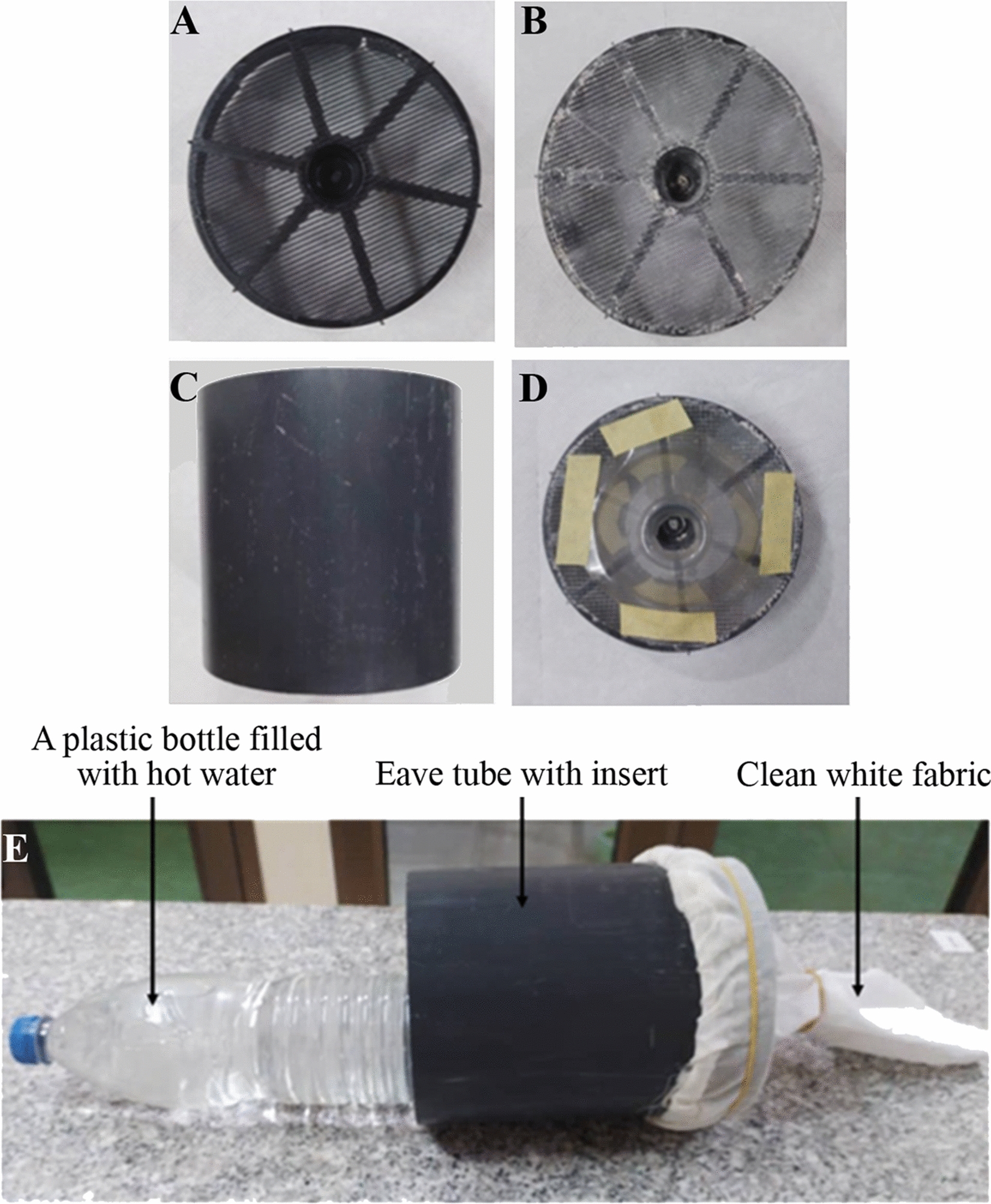
The eave tube components and eave tube bioassay device. **A** Untreated eave tube insert.** B** Treated insert with visible 10% beta-cyfluthrin powder. **C** 20-cm tube of PVC. **D** Treated insert with WHO cone fixed with a rubber band to hold it in place and prevent mosquito escape. **E** Insert at the end of a dark pipe; the opposite side contains a clean plastic bottle filled with hot water with a smelly sock at the end to attract mosquitoes

The eave tube assay was described in detail in a previous study [22]. Briefly, it consists of a black 20-cm PVC pipe (Fig. 1c), in which a plastic disc containing a 10% beta-cyfluthrin-treated insert is fixed (Fig. 1b) and a clean white sheet wrapped on one end (Fig. 1e). On the other end of the PVC, a clean plastic bottle filled with hot water (1.5 l) with the end wrapped in a smelly sock to provide host cue is inserted across it (Fig. 1b). A total of 15 mosquitoes in replicates of four (60 females) were released through the white sheet and allowed to interact with the treated insert for 1 h, and then mortality was scored after 24 h holding of mosquitoes in cages supplied with honey. Tests were repeated once every month on the same date with 10% beta-cyfluthrin-treated inserts kept in open air to assess residual activity over 9 months. Shorter exposure periods (30 s, 1 min, 2 min) were tested to assess the effect of treated inserts on transient exposure to mosquitoes.

### Bio-efficacy of long-lasting insecticidal nets (LLINs) versus insecticide-treated inserts

Classic bioassays were performed with PermaNet 2.0 LLINs in WHO cones with 3 min exposure [26] versus cone exposure of mosquitoes for 3 min to 10% beta-cyfluthrin-treated inserts (Fig. 4). Five females in replicates of 10 (50 females) of susceptible and wild resistant *Cx. quinquefasciatus* were tested.

### Semi-field studies

**Fig. 2:**
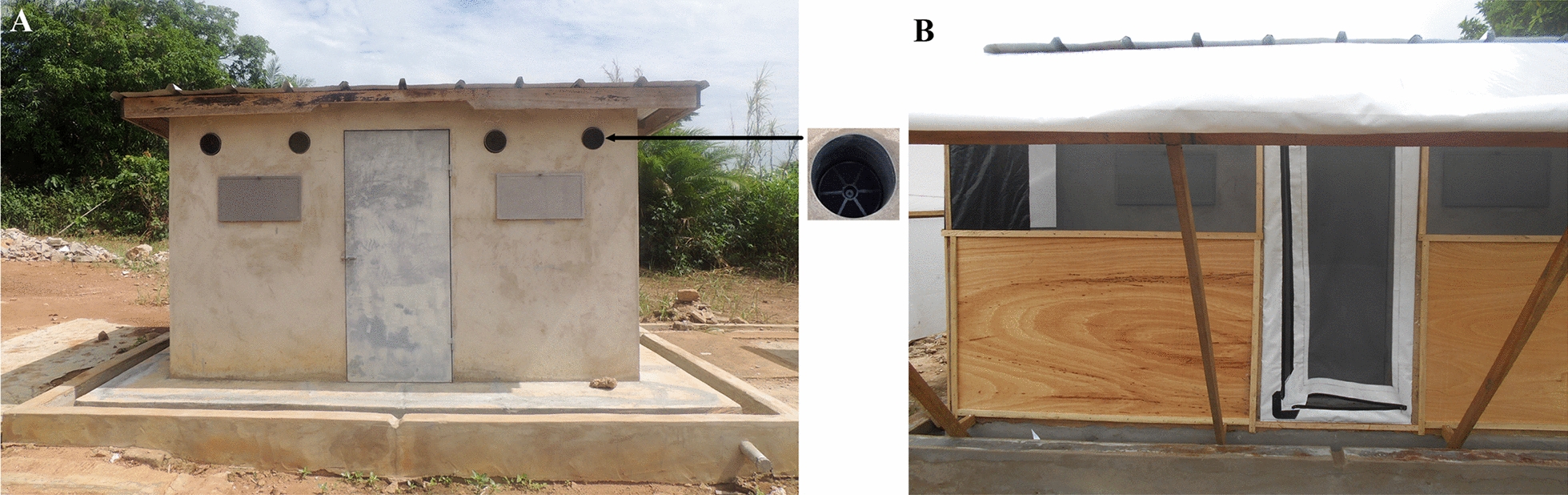
Experimental hut with modifications. **A **Experimental hut fitted with eave tubes. **B** Experimental hut with enclosure and fitted with eave tubes

Large enclosures erected around experimental huts fitted with eave tubes were used for release–recapture of resistant *Cx. quinquefasciatus.* The experiment was conducted at the M’bé experimental hut station from June to July 2018 using two experimental huts constructed in West African design as described previously [22]. The huts are 3.25 m long, 1.76 m wide, and 2 m high [27, 28]. The interior walls of the huts are made of concrete brick, with a corrugated iron roof and a solid base with a water-filled moat to protect against ants. For previous experiments against malaria vectors, 12 holes were drilled at the eave level on three sides of the hut to fit eave tubes and inserts freshly treated with 10% beta-cyfluthrin, but for the current study, half of the openings were blocked, and the remaining six holes (two holes on each side) were used (Fig. 2a). An enclosure was erected around the huts to allow for the recapture of mosquitoes after contact with the eave tube inserts (Fig. 2b).

Two sets of experiments were conducted to (i) evaluate the entry preference of *Cx. quinquefasciatus* via windows or eaves, and (ii) evaluate the impact of insecticide-treated inserts against the proportion of resistant *Cx. quinquefasciatus* collecting in the hut. In the first experiment, approximately 200 female mosquitoes were released per night up to eight replicates, and in the second experiment 200 mosquitoes per night in replicates of six were released.

### Statistical analysis

Data analyses were performed using R version 4.0.3 software. The resistance status of the wild *Cx. quinquefasciatus* was assessed experimentally using the WHO cylinder test, and the results obtained were analysed according to the WHO criteria [29]; odds ratios (OR) were used to assess the effect of pre-exposure to PBO on pyrethroid mortality rates. For the cone test and the eave tube short contact assays, the proportions of mosquitoes killed were analysed with respect to the treatment and the exposure time (for the eave tube short contact assay only) as explanatory variables, using the Chi-square test followed by multiple comparisons using the fisher.multcomp function of the package RVAideMemoire.

Residual activity of eave tube inserts was then monitored for 9 months using the eave tube bioassay, and the mosquito mortality data were fitted with a generalized linear model with binomial distribution (GLM), using the function ‘glm’ from the R base package. Interactions between insecticide and persistence intervals (time since treatment) were also included in the GLMs. Pairwise comparisons were performed with the final model using the ‘multcomp’ package in R. For overnight release–recapture experiments, the number of *Cx. quinquefasciatus* entering huts through the eaves or windows was analysed using generalized linear mixed models (GLMMs) with a binomial distribution, and the treatment (open eaves or windows) was included as independent variable. The night of capture and sleepers were considered as random effects. Similarly, the number of *Cx. quinquefasciatus* entering huts with untreated or insecticide-treated inserts was analysed using a GLMM with a binomial distribution, considering the treatment (treated or untreated insert) as independent variable and the night of capture and sleepers as random effects. Odds ratios were included to compare models with or without treated inserts.

## Results

### Insecticide susceptibility test

The mortality rate with all insecticides was 100% against susceptible *Cx. quinquefasciatus*, indicating the good quality of the insecticide-impregnated papers (data not shown). Figure 4 shows the insecticide resistance status of wild *Cx. quinquefasciatus* from Bouaké to the insecticides tested. The mortality rates were very low for all pyrethroids used and for dichlorodiphenyltrichloroethane (DDT) (range: 0.95–4.63%). The rates were moderately higher for bendiocarb (26.0%) and pirimiphos-methyl (41.0%).

**Fig. 3:**
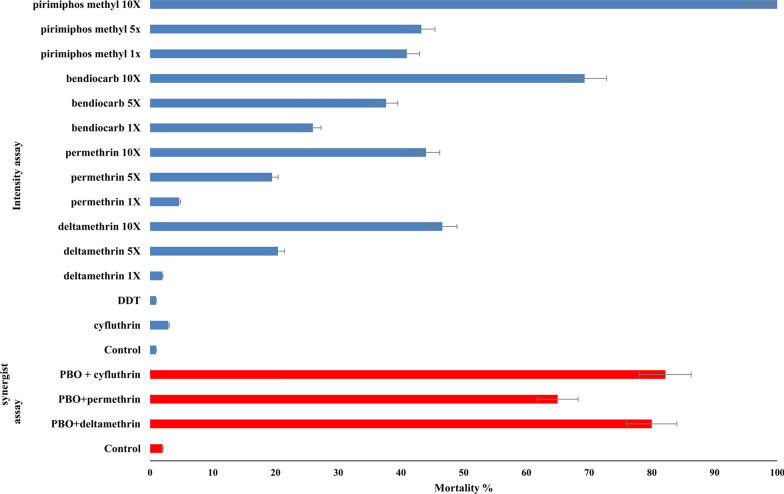
Percentage mortality in insecticide-resistant *Cx. quinquefasciatus* from Bouaké in WHO cylinder bioassays. Blue and red bars represent intensity and synergist assay, respectively. Error bars represent 95% confidence intervals

The intensity assays with 5× and 10× DDs with all insecticides produced mortality rates no greater than 50% except for 10× pirimiphos-methyl DD (100%), which was significantly higher compared to pirimiphos-methyl DD (*χ*^2^ = 83.19, *df* = 1,* P* < 0.0001), indicating severe resistance levels to pyrethroids and DDT in *Cx. quinquefasciatus* from Bouaké. Nevertheless, pre-exposure to PBO boosted the activity of the pyrethroids to cyfluthrin from 2.94 to 82% (OR = 27.94, CI 12.79–151.4, *P* < 0.0001), deltamethrin from 1.96 to 80.2% (OR = 40.89, CI 16.83–95.26, *P* < 0.0001), and permethrin from 4.63 to 65% (OR = 14.04, CI 0.71–27.36, *P* < 0.0001) (Fig. 3).

### Bio-efficacy of LLINs versus insecticide-treated inserts

**Fig. 4:**
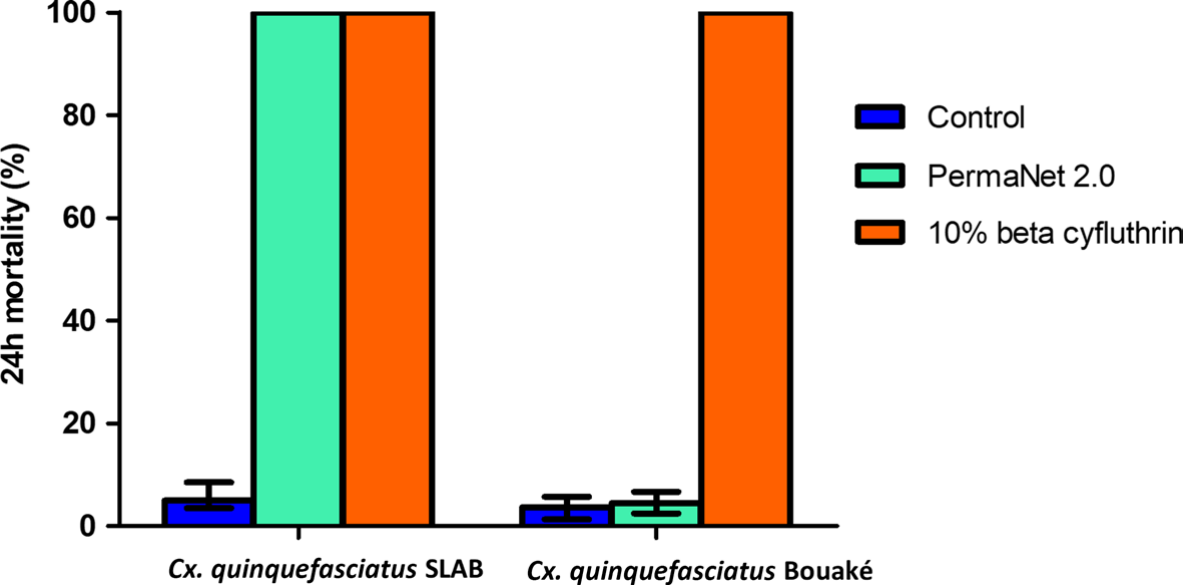
Percentage mortality from WHO cone assay with PermaNet 2.0 LLINs versus 10% beta-cyfluthrin-treated inserts of *Cx. quinquefasciatus* susceptible SLAB and resistant Bouaké strains. Error bars represent 95% confidence intervals

Exposure for 3 min of susceptible *Cx. quinquefasciatus* SLAB on PermaNet 2.0 induced high mortality rates (100%) compared to those induced by 10% beta-cyfluthrin-treated inserts (100%). By contrast, the percentage mortality rates of the wild *Cx. quinquefasciatus* on PermaNet 2.0 were very low, at 4.5%, whereas the rate was still 100% on 10% beta-cyfluthrin-treated inserts (*χ*^*2*^ = 249.82, *df* = 1, *P* < 0.001) (Fig. 4).

### Short contact assays

The mortality of the resistant *Cx. quinquefasciatus* increased with this exposure time. Spending 30 s on 10% beta-cyfluthrin-treated inserts killed half of the exposed individuals, whereas exposure for 1 min killed 88% (*χ*^*2*^ = 11.59, *df* = 1, *P* < 0.0006) and exposure for 2 min achieved 100% mortality, but the difference between 1 min and 2 min was not significant (*X*^*2*^ = 0.27, *df* = 1, *P* = 0.60) (Table 1).

**Table 1:** Percentage mortality in insecticide-resistant *Cx. quinquefasciatus* from Bouaké following shorter exposure period (30 s, 1 min, 2 min**)**

Strain	Treatment	Exposed time	N	Mortality % [CI 95]
Bouaké	Control	30 s	52	0
Bouaké	10% beta-cyfluthrin	30 s	52	44^a^ [31.3–57.8]
Bouaké	Control	1 min	57	0
Bouaké	10% beta-cyfluthrin	1 min	59	88^a^ [78.2–94.5]
Bouaké	Control	2 min	58	0
Bouaké	10% beta-cyfluthrin	2 min	56	100^b^

Values in the same column not sharing a letter superscript differ significantly (*P* < 0.05, GLMMs) [CI 95] 95% confidence intervals

### Residual monitoring of 10% beta-cyfluthrin activity on inserts

**Fig. 5:**
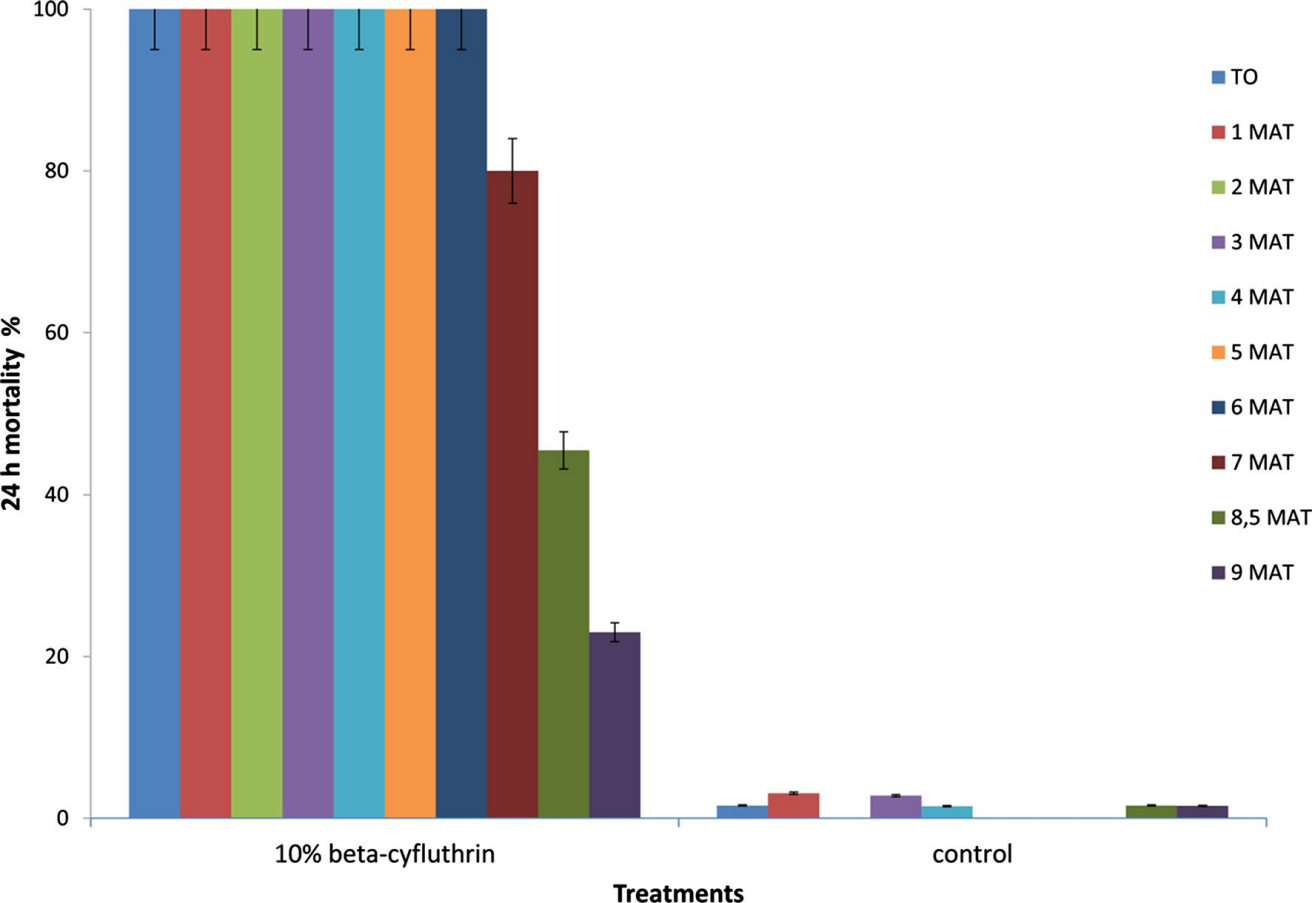
Residual activity of 10% beta-cyfluthrin-treated insert against insecticide-resistant *Cx. quinquefasciatus* from Bouaké. MAT months after treatment. Error bars represent 95% confidence intervals

On inserts, 10% beta-cyfluthrin killed 100% at T0 (freshly treated inserts) continuously for 6 months; at 7 months the mortality rate was significant (80%) (OR = 9.73, CI 0.81–1165.06, *P* = 0.89) on wild pyrethroid-resistant *Cx. quinquefasciatus*. However, the mortality rate declined to under 50% at 8 months, and 23% at 9 months (OR = 459.06, CI 4.53–1464.09, *P* = 0.0011) on wild pyrethroid-resistant *Cx. quinquefasciatus* (Fig. 5).

### Semi-field studies: overnight release and recapture

#### *Experiment 1*: *Cx. quinquefasciatus* entry preference

**Fig. 6:**
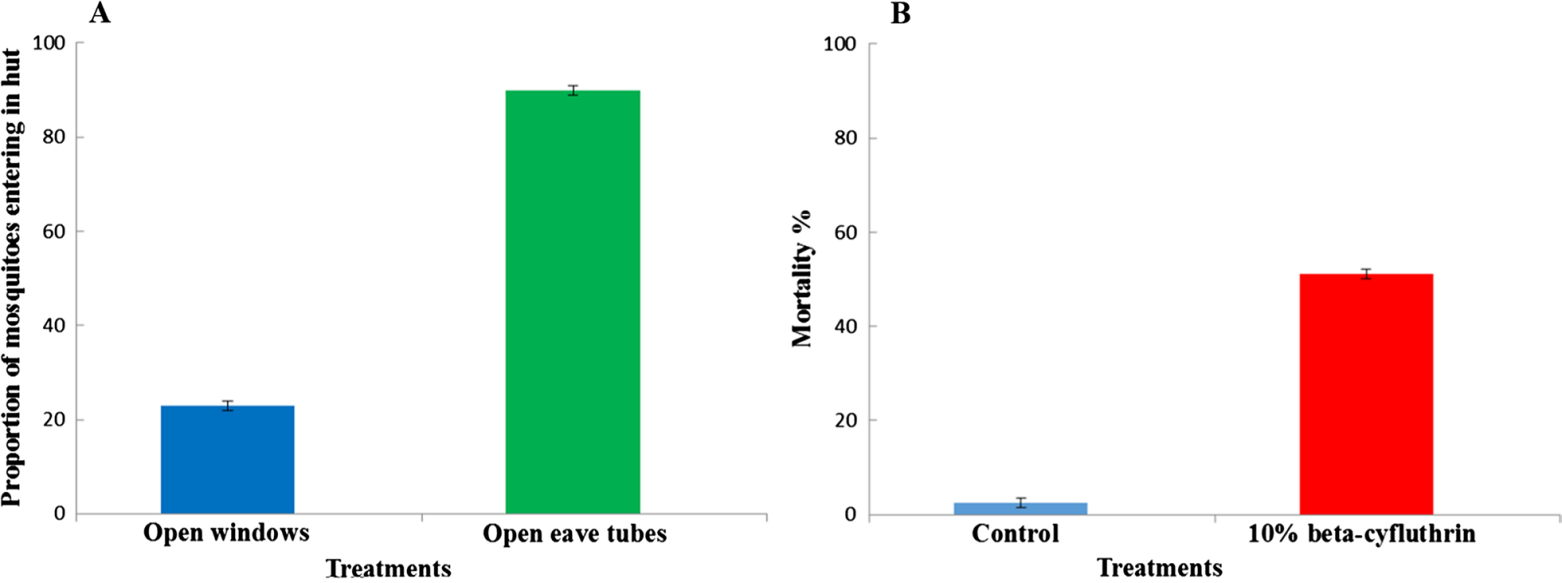
Experimental hut evaluations. **A** Collection of *Cx. quinquefasciatus* from Bouaké to the hut via open windows or eave tubes within enclosure around huts.** B** Percentage mortality of insecticide-treated inserts against the proportion of resistant *Cx. quinquefasciatus* from Bouaké collecting in the hut within the enclosure. Error bars represent 95% confidence intervals

Following eight nights of release and recapture, more than 90% of resistant *Cx. quinquefasciatus* released in the experimental enclosure entered the hut with open eaves overnight; the mean (±SE) of 90.08 ± 4.2 was significantly higher (*P* < 0.001) than that of the experimental enclosure with open windows (22%), at 22.88 ± 0.08 (Fig. 6a).

#### *Experiment 2: *impact of insecticide-treated inserts against the proportion of resistant *Cx. quinquefasciatus* collecting in the hut

The overnight mortality of wild *Cx. quinquefasciatus* that collected in the control hut (hut with untreated inserts) was below 5%. Treated inserts showed higher mortality (51%) against resistant pyrethroid *Cx. quinquefasciatus* (OR = 39.8, CI 23.3–68.2 *P* < 0.001) (Fig. 6b).

## Discussion

The bio-efficacy of eave tubes has been tested on several occasions against insecticide-resistant strains of *Anopheles* and *Culex* mosquitoes, but in the laboratory only. This is the first study in semi-field conditions that clearly demonstrated their impact against a wild population of highly resistant *Cx. quinquefasciatus* from Côte d’Ivoire. Susceptibility assays with the local *Cx. quinquefasciatus* confirmed the strong resistance against four classes of insecticides recommended in public health. One possible explanation for this intensive resistance in this species is the fact that adult *Anopheles* and *Culex* are found in sympatry within homes for blood meals, and therefore concurrently exposed to indoor vector control tools, mainly LLINs and indoor residual spraying (IRS) [30]. A previous study demonstrated higher insecticidal impact of eave tubes on various species of resistant mosquitoes [15], in which insecticide-treated inserts were able to induce far greater mortality against highly resistant *Culex* than did standard LLINs. Similar observations were made in our current study, with eave tubes killing 100% *Culex* versus only 4.5% by the standard LLIN. The large difference in activity may be due to the difference in delivery or bioavailability of the active ingredient (AI) between the PermaNet 2.0 LLIN, in which the AI is bound to the fibres through coating, and the insecticide-treated inserts in which the insecticide powder is directly deposited onto the insert surface [15]. In the present study, the monitoring of insecticide persistence showed that insecticide-treated inserts were effective against resistant *Cx. quinquefasciatus* for at least 9 months before a significant decline in activity. The trend in residual activity was similar to that observed with wild pyrethroid-resistant *An. gambiae*, although the decline after 9 months was faster with *Cx. Quinquefasciatus* [22]. A previous study in Cote d’Ivoire showed *Cx. quinquefasciatus* to be more strongly resistant than *An. gambiae* [8]. Further studies are needed to update the resistance status of *Cx. quinquefasciatus* from this area of Côte d’Ivoire, including the underlying defence mechanisms. The data from the release–recapture experiment conducted in experimental huts demonstrated good performance of 10% beta-cyfluthrin-treated inserts against resistant *Cx. quinquefasciatus.* Insecticide-treated inserts killed half (51%) of pyrethroid-resistant *Cx. quinquefasciatus* released within the enclosure, which is similar to the proportion of resistant *An. gambiae* killed (= 55%) with the same treatment within the enclosure at the same site [22]. Also, we demonstrated that *Cx. quinquefasciatus* released in the experimental enclosure preferred entering through open eaves. Previous studies showed that the preference for open eaves was about the same with *Culex *spp. and other disease-transmitting and nuisance biting mosquito species [21]. The nuisance caused by *Culex* determines the use of insecticide-treated nets (ITN), and people will lose interest in the use of ITNs when they fail to protect users from bites of resistant *Culex* species [30]. In the present study, 90% of host-seeking mosquitoes entered huts via eave tubes, whereby half of them were killed. This indicates that the eave tube approach could be an important component for improving overall well-being and ensuring healthy lives rather than serving merely as a malaria vector control tool [18].

A limiting factor in the current study is that it did not provide data on *Cx. quinquefasciatus* life history traits (survival, fecundity) after exposure to sublethal doses. The observation was made within 24 h only post-exposure, ignoring the impact behind the screen.

## Conclusions

Eave tube technology is a novel method for delivery of insecticide to the house. It attracts nuisance host-seeking *Cx. quinquefasciatus* mosquitoes and is as effective in controlling them as its efficacy against pyrethroid-resistant *An. gambiae*, despite the high level of resistance *Cx. quinquefasciatus* have developed. House improvement through lethal lure devices such as eave tubes could be employed as an integrated mosquito control intervention.

## Data Availability

The datasets used and/or analysed during the current study are available from the corresponding author on reasonable request.

## Abbreviations

LLIN: Long-lasting insecticidal net
ITN: Insecticide-treated net
PBO: Piperonyl butoxide
IRS: Indoor residual spraying
DDT: Dichlorodiphenyltrichloroethane
